# Relapse risk after FLAMSA RIC based allogeneic hematopoietic stem cell transplantation in heavily pretreated AML patients

**DOI:** 10.1038/s41409-026-02960-8

**Published:** 2026-07-15

**Authors:** Jule Ussmann, Madlen Jentzsch, Sebastian Schwind, Marco Herling, Vladan Vučinić, Maximilian Merz, Inken Hilgendorf, Andreas Hochhaus, Uwe Platzbecker, Klaus H. Metzeler, Georg-Nikolaus Franke

**Affiliations:** 1 Department for Hematology, Cell Therapy, Hemostaseology and Infectious Diseases, University of Leipzig Medical Center, Leipzig, Germany; 2 CCCG, Cancer Center Central Germany, Leipzig/Jena, Germany; 3 Hematology/Oncology, Jena University Hospital, Jena, Germany; 4 University Hospital of Dresden, Dresden, Germany

**Keywords:** Acute myeloid leukaemia, Risk factors, Medical research

Despite recent advances in the treatment of acute myeloid leukemia (AML), allogeneic hematopoietic stem cell transplantation (allo-HSCT) remains the only potentially curative treatment for many patients with high-risk AML [1]. Unfortunately, up to 30% of AML patients are primary refractory, and up to 46% of AML patients experience early relapse within 6 months after treatment completion [2]. For these patients, Schmid et al. explored an approach to improve outcomes by combining chemotherapy with fludarabine, amsacrine and cytarabine (FLAMSA) with a consecutive reduced intensity conditioning (RIC) allo-HSCT shortly after the end of cytoreductive chemotherapy followed by rapid tapering of immunosuppression and prophylactic donor lymphocyte infusions (DLI) in patients who did not develop a Graft-versus-Host Disease (GvHD) [3]. With 2-year overall survival (OS) rates of 42% and event-free survival (EFS) of 40%, this regimen is an established approach in high-risk AML patients [1]. However, the term high-risk AML is not well defined and changed with new disease classifications. The initial study as well as follow up trials included significant numbers of high-risk AML patients (defined by cytogenetics or delayed treatment-response) who were in morphological remission at the time of allo-HSCT [4–6]. Studies including mainly patients with active AML undergoing FLAMSA RIC do not solely focus on highly treatment-refractory patients [7, 8].

With our study, we aimed to evaluate outcomes and risk-factors in an intensively pretreated cohort of relapsed/refractory (r/r) patients who had active disease and retrospectively analyzed 106 patients (median age 55, range 21–73 years, 51% female, also view Supplementary Material Table S1), who underwent FLAMSA RIC based allo-HSCT at the university hospitals of Leipzig and Jena between 2006 and 2022. All had active AML at the time of allo-HSCT (i.e. blast count in bone marrow ≥5% and/or persistent blasts in peripheral blood before FLAMSA RIC).

Median EFS and OS were 134 (range 1–3115) days and 249 (range 1–3327), respectively. At two years, EFS and OS were 15% and 20%, respectively (Fig. 1A). While typical prognostic factors were not associated with outcome (Table S2), the leukemic burden in bone marrow prior to FLAMSA RIC associated with outcomes: patients with blastcounts in bone marrow above the median of the whole population (i.e.16%) had a significantly shorter EFS (*P* = 0.02, Fig. S1A) and OS (*P* = 0.02, Fig. S1B) compared to patients with ≤16% blasts. The competing risk analysis showed a higher non-relapse mortality (NRM) for patients with ≤6 months from diagnosis to allo-HSCT compared to patients with an interval >6 months (*P* = .02; Fig. S2A), but no associations with EFS (*P* = 0.81) and OS (*P* = 0.88). Patients who had received ≥3 prior lines of treatment had a higher cumulative incidence of relapse (CIR) compared to patients with <3 prior lines of treatment (*P* = 0.01, Fig. S2B), however, prior treatment did not significantly associate with EFS (*P* = 0.23) and OS (*P* = 0.51, Table S2).

**Fig. 1:**
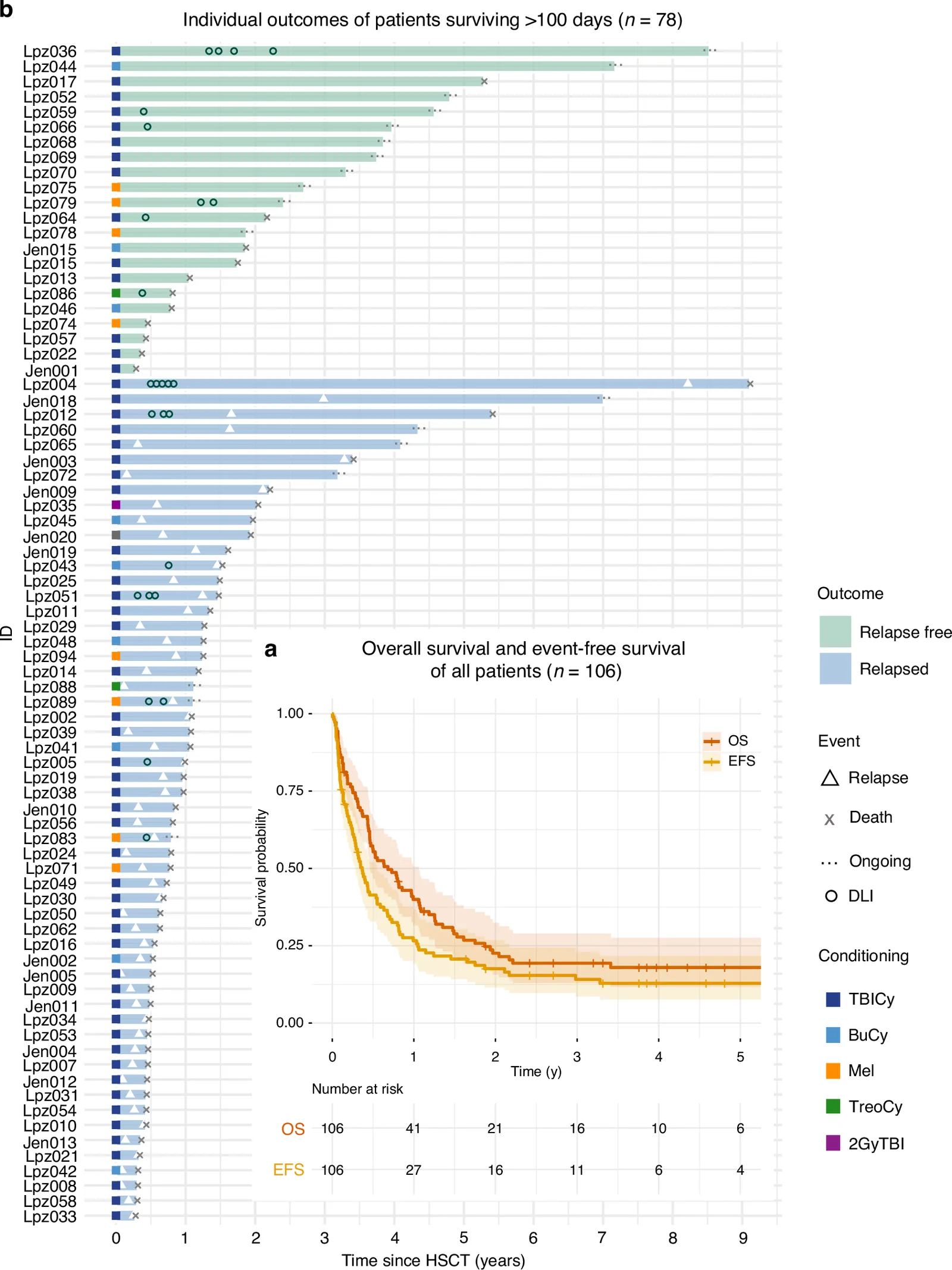
Survival after FLAMSA RIC based allo-HSCT in our cohort. **a** Kaplan-Meier estimates for overall survival and event-free survival of all patients (*n* = 106) and **b** Individual outcome of patients surviving > 100 days (*n* = 78): each bar represents the survival time of one of the 78 individual patients who survived day 100 post allo-HSCT. Patients are grouped according to their remission status at the timepoint of last observation. Represented in green: patients who remained relapse-free, in blue: patients who relapsed eventually. Relapse and death are marked on the bars as displayed in the legend. An arrow symbolizes an ongoing observation at data censoring. Conditioning regimens are displayed by colored rectangles at the beginning of each bar. BuCy busulfan and cyclophosphamide conditioning, DLI donor lymphocyte infusion, EFS event-free survival, Gy Gray, Mel melphalan conditioning, OS overall survival, TBICy total body irradiation and cyclophosphamide conditioning, TreoCy treosulfan and cyclophosphamide conditioning.

In multivariate analysis, the threshold of 16% blasts in bone marrow prior to FLAMSA RIC retained its predictive value for EFS and OS, however—as in univariate analysis—none of the other tested variables were significantly associated with OS or EFS (Table S3). Chronic GvHD was documented for 17 patients and 13 patients received at least one prophylactic DLI after allo-HSCT (Fig. 1B). In landmark analyses, neither the development of a chronic GvHD (Table S2) nor prophylactic DLI (Table S3) associated with EFS or OS. Since the GvL effect needs time to be effective, the missing benefit of chronic GvHD and DLI in our cohort might be due to their especially strong competitors relapse and NRM.

Till last follow up, 30% of the patients had not experienced relapse. Compared to patients, who eventually relapsed after FLAMSA RIC based allo-HSCT, they were significantly more often transplanted for a primary refractory AML (*P* = 0.05), had received less pretreatment ( < 3 vs. ≥ 3 lines, *P* < 0.01), had a shorter time interval from diagnosis to allo-HSCT (median 3.5 [range 0–45] months; *P* = 0.01) and had lower blast counts prior to allo-HSCT (in blood *P* = 0.02 and bone marrow *P* = 0.04; Table S1). At day 28 after allo-HSCT, 56 patients achieved blast clearance, but this was not predictive for outcomes which were comparable (MLFS/CRc *vs*. blast persistence: EFS, *P* = 0.55; OS, *P* = 0.86 Fig. S3A-B).

The CD34+ chimerism in bone marrow predicted a relapse within the next 14 and 28 days with an area under the curve of 0.83 and 0.81, respectively, which declined rapidly for longer time periods (Fig. S5).

In our cohort, the leukemic burden prior to allo-HSCT was the only factor influencing outcomes. Similarly, Sockel et al. found an association of >20% blasts prior to allo-HSCT after melphalan-based conditioning with shorter EFS and OS especially in relapsed patients [9].

Our patients were treated heterogeneously prior to FLAMSA RIC but the amount of prior therapy was of relevance in our study. Patients who lived progression-free after FLAMSA RIC based allo-HSCT had received less pretreatment and had shorter intervals from diagnosis to allo-HSCT. These aspects are underlined by recently published data of the ASAP trial, randomizing patients with AML refractory to first induction or relapse to either salvage therapy to achieve CR prior to allo-HSCT or immediate allo-HSCT after sequential conditioning consisting of intensive chemotherapy followed by RIC [10]. The study showed no survival benefit for the salvage therapy prior to allo-HSCT compared to the sequential approach.

Our competing risk analyses showed a higher CIR in heavily pretreated patients ( ≥ 3 lines) and a higher NRM only in patients, who went from diagnosis to allo-HSCT in <6 months. This is most likely due to the many highest-risk patients in our cohort not responding to (multiple) lines of intensive chemotherapy, thus reflecting a high-risk disease biology. Unfortunately, due to the retrospective character of the study and the long period analyzed, we do not have a comprehensive molecular characterization of our patient set. Correspondingly, typical risk factors in AML and allo-HSCT did not show associations with outcomes in our analysis.

In this high-risk population, early prediction of imminent relapse allowing preemptive therapy remains a major challenge. Remission status at day 28 did not associate significantly with long-term OS and EFS, and although a drop in CD34+ chimerism predicted a relapse, it was not possible to define an exact cut-off at day 28 and day 100 after allo-HSCT. A combination of different methods (chimerism, molecular data and flow cytometry) might enable to identify candidates for preemptive treatment.

With 106 patients, to our knowledge, this is one of the largest studies analyzing FLAMSA RIC based allo-HSCT in active r/r AML patients. The coherent patient collective regarding the disease status is one of its strengths, as is the long follow-up period. However, changes regarding pretreatment, RIC regimens and prophylactic medication over time bring a certain heterogeneity.

In conclusion, in patients with active disease, relapse rate is high, and median survival times are exceptionally short compared to outcomes after allo-HSCT in other patient populations. While keeping in mind the high aggressiveness of the underlying diseases and the heavily pre-treatment in our cohort, FLAMSA RIC based allo-HSCT was able to lead to long-term survival in some of our patients (Fig. 1a). But the short survival and high relapse incidence emphasize the need to carefully weigh curative *vs*. palliative treatment options for these patients.

## Supplementary information

Supplemental Material

## Data Availability

Data is available from the corresponding author upon reasonable request
