# Supplementary material for: Relapse risk after FLAMSA RIC based allogeneic hematopoietic stem cell transplantation in heavily pretreated AML patients
Source: Bone Marrow Transplant. 2026 Jul 15;61(9):1257–9. doi: 10.1038/s41409-026-02960-8 (PMC13569441; doi:10.1038/s41409-026-02960-8)
Supplement: Supplementary file 1 [file 41409_2026_2960_MOESM1_ESM.docx]

**Supplementary Information to**

**Relapse risk after FLAMSA RIC based allogeneic hematopoietic stem cell transplantation in heavily pretreated AML patients**

*Ussmann et al.*

## ***Methods***

*Patient’s characteristics*

Patients were stratified into risk groups by cytogenetics at diagnosis (available for 96 patients) according to the Revised Medical Research Council (MRC) prognostic classification ^1^. ELN2022 risk classification was applicable in 66 patients at diagnosis ^2^. Median time from diagnosis to allo-HSCT was six (range 0-45) months. One treatment line was defined as one or more cycles of a certain treatment regimen applied to induce or consolidate a remission or as salvage treatment, a prior allo-HSCT was also counted as one treatment line. Hematopoietic cell transplantation-specific comorbidity index (HCT-CI) was calculated up to 28 days prior to allo-HSCT ^3^. To determine disease status prior to allo-HSCT, bone marrow puncture was performed up to 28 days prior to allo-HSCT (before administration of FLAMSA). Detailed patients’ and transplant characteristics are summarized in Supplementary Table S1. The median follow-up alive after allo-HSCT was 46 months.

*Human leucocyte antigen-Typing and donor selection*

High resolution molecular human leukocyte antigen (HLA)-typing of HLA-A, HLA-B, HLA-C, HLA-DRB1, HLA-DQB1 was performed for all patients and all unrelated and haploidentical donors. Matched sibling donors (MSD) were at least identical at a low resolution typing for HLA-A, -B, -C, and at high resolution for HLA-DRB1 and -DQB1. A 10/10 matched unrelated donor (MUD) was available for 66 (62%) patients, 28 (26%) had a mismatch unrelated donor (MMUD), 9 (8%) patients had an MSD, and three (3%) received an allo-HSCT from a haploidentical donor.

A high-risk constellation for cytomegalovirus (CMV) was defined as allo-HSCT of a serological CMV-negative donor in a serological CMV-positive recipient.

## *FLAMSA and Conditioning*

All patients received a cytoreductive chemotherapy with fludarabine, amsacrine and cytarabine on 4 consecutive days ^4^.

Sixty-eight patients received conditioning as initially published by Schmid *et al.* ^4^. The FLAMSA part (*i.e.* fludarabine 30 mg/m^2^, amsacrine 100 mg/m^2^ and cytarabine 2000 mg/m^2^) was administered on days -12 to -9. After three days of pause, patients received 4 Gy total body irradiation (TBI) at day -5 prior to HSCT in one setting. On the following two days, cyclophosphamide at a dose of 40 mg/kg body weight (BW) was administered.

16 patients received conditioning with busulfan (4 x 0.8 mg/kg BW/day on two consecutive days) instead of TBI and cyclophosphamide.

13 patients received conditioning with melphalan only as published by Saure *et al*. ^5^. The four-day FLAMSA part started as of day -9 prior to allo-HSCT. Two days prior to allo-HSCT, patients received a single Melphalan dose of either 200 mg/ m^2^ for patients with an age <50 years, 150 mg/ m^2^, for patients aged between 50 and 60 years, or 100 mg/ m^2^, for patients aged 60 and older. Six patients received treosulfan based conditioning according to Holtick *et al.* ^6^ on three consecutive days starting 6 days prior to allo-HSCT at a dose of 10 g/m^2^ per day followed by 60 mg/kg BW cyclophosphamide at day -3 and -2 prior to HSCT. 2 patients received TBI with 2 Gy only as conditioning between FLAMSA and allo-HSCT ^7^. The type of conditioning regimen was not associated with overall survival (OS, Figure S4A) or event-free survival (EFS, Figure S4B).

## *Graft-versus-Host Disease prophylaxis*

All patients except those with haplo-identical donors received a prophylaxis for Graft-versus-Host Disease (GvHD) with antithymocyte globuline (ATG) Fresenius/Neovii, cyclosporine A (CsA) and mycophenolate mofetil (MMF). ATG was administered intravenously at a dose of 20 mg/kg BW per day for patients with human leucocyte antigen (HLA) mismatches or 10mg/kg BW per day for patients with matched donors on 3 consecutive days shortly prior to HSCT. CsA was started i.v. on day -1 prior to allo-HSCT at a dose of 5 mg/kg BW twice daily to obtain initial trough serum levels of 120-150 ng/ml and tapered as of day 60 after HSCT with the intention to end immunosuppressants at day 90 post allo-HSCT. Patients with haploidentical did not receive ATG but post transplantation cyclophosphamide at a dose of 50 mg/kg BW on days 3 and 4 post HSCT. Instead of CsA, patients received tacrolismus as of day 5 post allo-HSCT. As in case of immunosuppression with CsA, tapering started at day 60 and intended end was at day 90 post HSCT. All patients additionally received MMF 2 g per day in two daily doses, starting on day 0 (day 5 in case of haploidentical donor). MMF was tapered between day 30 and 50 post HSCT and discontinued on day 50.

*Evaluation of remission and engraftment*

After allo-HSCT, first engraftment was defined as the presence of ≥1 Gpt/l leukocytes and ≥50 Gpt/l platelets (transfusion independent) in peripheral blood. Median time to leukocyte and platelet engraftment was 14 (range 8-49) and 15.5 (range 10-97) days, respectively. Morphologic remission and chimerism in bone marrow were evaluated regularly. Morphologic remission in bone marrow was evaluated according to ELN2022 recommendations ^8^.

After HSCT, we included data of bone marrow examinations at day 28 ± 7 into our analysis, for remission evaluation at the second timepoint after HSCT, we included bone marrow evaluations at day 100 ± 28. To determine outcome associations with disease status at these two timepoints, we performed landmark analysis by calculating the OS and EFS times starting from the timepoint of bone marrow puncture, to minimize time-dependent bias.

For the evaluation of donor chimerism in bone marrow, Short Tandem Repeat (STR) markers were measured by quantitative polymerase chain reaction either on all cells, or on flow cytometry sorted CD34+ cells. At day 28 ± 7, total chimerism and CD34+ based chimerism in bone marrow were available for 82 patients (95% of all patients with bone marrow analysis available at the timepoint) and 41 patients (48%), respectively. At day 100 ± 28, total chimerism and CD34+ based chimerism in bone marrow were available for 29 (48%) patients and 23 (38%) patients, respectively. Comparing patients with a total bone marrow chimerism ≥ 98% *vs.* < 98% (the threshold used in various previous studies ^6,9^) showed no association with OS or EFS in patients who had any kind of remission (PR, MLFS, CRi, CR) at the time of chimerism analysis, neither did other thresholds such as the median total bone marrow chimerism of 100%, or 93% calculated with the R package “OptimalCutpoints” ^10^. Of note, in one of the above-mentioned studies, 39 of our patients had been included ^9^. Their data was renewed for our analysis and after the expansion of the cohort, the chimerism threshold was not prognostic anymore. Also, the comparison of patients (in remission at the timepoint of bone marrow chimerism analysis) with bone marrow chimerism of CD34+ cells above and below these thresholds did not show associations with OS and EFS. However, we performed c-statistics to test the predictive value of bone marrow chimerism at different timepoints (i.e. 84, 56, 28, 14 days) before relapse occurred. Total bone marrow chimerism was not predictive for relapse (Figure S5).

*Donor-Lymphocyte Infusion and Graft-versus-Host Disease*

As performed in the initial study protocol ^4^, donor lymphocyte infusions (DLI) were administered from day +120 after HSCT, or at least 30 days after end of immunosuppressive treatment if no signs of GvHD were evident. Starting dose in our cohort was 1 or 5 x 10^5^ CD3+cells/kg of recipient BW, doses were increased over up to a maximum of 5 x 10^7^ CD3+cells/kg recipient BW.

Engraftment was required to diagnose GvHD. Acute GvHD was evaluated according to Mount Sinai Acute GvHD International Consortium (MAGIC) criteria ^11^

Chronic GvHD was registered as present if its evidence was documented at any time >100 days after allo-HSCT. For chronic GvHD, accurate data concerning onset, extent and treatment was mostly missing due to our file-based analysis, and we did no further grading and only evaluated the occurrence. In cases of locally uncontrolled or progressive GvHD, immunosuppression was either prolonged or extended using systemic steroids or other immunosuppressants.

Median duration of immunosuppressive therapy was 109 days (range 1-1064 days) for the whole cohort. In a total of 13 patients, a median of 1.5 DLI (range 1-5) were administered and the median time from allo-HSCT to first prophylactic DLI was 165 (range 113-488) days. In a landmark analysis at day 165 after allo-HSCT, prophylactic DLI did neither associate with OS (*P*=.17), nor EFS (*P*=.42; Figure S7).

Acute GvHD ≥°2 was diagnosed in 28 patients who then received systemic treatment. Neither the development of acute GvHD (OS *P*=1.0; EFS *P*=.53) nor chronic GvHD (landmark analysis at day 100; OS *P*=.18; EFS *P*=.24) did significantly associate with outcomes.

*Statistical analysis*

OS was defined as time from allo-HSCT to death of any cause. EFS was defined as time from allo-HSCT to relapse or death from any cause. For unknown outcomes, patients were censored at the time of last follow up. We analyzed OS and EFS in a univariate analysis by Kaplan-Meier method combined with Logrank-testing. For baseline-variables, non-relapse mortality (NRM, defined as death without relapse) and cumulative incidence of relapse (CIR) were calculated as competing events after allo-HSCT using the Fine and Gray method ^12^.

Comparing variables, we used Fisher’s exact test for categorical variables and Wilcoxon Rank-Sum test for continuous variables. Receiver-operating characteristics (ROC) curves were used to evaluate the strength of relapse prediction with chimerism. The multivariate analysis was performed as COX-regression model including all variables with a *P*-value <0.2 in univariate analysis (Table S2 and Table S3). The statistical analyses were all performed using the statistical software R Studio (R version 4.1.2) ^13^**.**

## **References**

1	Grimwade D, Hills RK, Moorman A V., Walker H, Chatters S, Goldstone AH *et al.* Refinement of cytogenetic classification in acute myeloid leukemia: Determination of prognostic significance of rare recurring chromosomal abnormalities among 5876 younger adult patients treated in the United Kingdom Medical Research Council trials. *Blood* 2010; **116**: 354–365.

2	Döhner H, Wei AH, Appelbaum FR, Craddock C, DiNardo CD, Dombret H *et al.* Diagnosis and management of AML in adults: 2022 recommendations from an international expert panel on behalf of the ELN. *Blood* 2022; **140**. doi:10.1182/blood.2022016867.

3	Sorror ML, Maris MB, Storb R, Baron F, Sandmaier BM, Maloney DG *et al.* Hematopoietic cell transplantation (HCT)-specific comorbidity index: A new tool for risk assessment before allogeneic HCT. *Blood* 2005; **106**. doi:10.1182/blood-2005-05-2004.

4	Schmid C, Schleuning M, Ledderose G, Tischer J, Kolb HJ. Sequential regimen of chemotherapy, reduced-intensity conditioning for allogeneic stem-cell transplantation, and prophylactic donor lymphocyte transfusion in high-risk acute myeloid leukemia and myelodysplastic syndrome. *Journal of Clinical Oncology* 2005; **23**: 5675–5687.

5	Saure C, Schroeder T, Zohren F, Groten A, Bruns I, Czibere A *et al.* Upfront Allogeneic Blood Stem Cell Transplantation for Patients with High-Risk Myelodysplastic Syndromeor Secondary Acute Myeloid Leukemia Usinga FLAMSA-Based High-Dose Sequential Conditioning Regimen. *Biology of Blood and Marrow Transplantation* 2012; **18**: 466–472.

6	Holtick U, Herling M, Pflug N, Chakupurakal G, Leitzke S, Wolf D *et al.* Similar outcome after allogeneic stem cell transplantation with a modified FLAMSA conditioning protocol substituting 4 Gy TBI with treosulfan in an elderly population with high-risk AML. *Ann Hematol* 2017; **96**. doi:10.1007/s00277-016-2887-4.

7	Hegenbart U, Niederwieser D, Sandmaier BM, Maris MB, Shizuru JA, Greinix H *et al.* Treatment for acute myelogenous leukemia by low-dose, total-body, irradiation-based conditioning and hematopoietic cell transplantation from related and unrelated donors. *Journal of Clinical Oncology* 2006; **24**: 444–453.

8	Döhner H, Wei AH, Appelbaum FR, Craddock C, DiNardo CD, Dombret H *et al.* Diagnosis and Management of Acute Myeloid Leukemia in Adults: 2022 Recommendations from an International Expert Panel, on Behalf of the European LeukemiaNet. Blood. 2022; **129**.

9	Pfrepper C, Klink A, Behre G, Schenk T, Franke GN, Jentzsch M *et al.* Risk factors for outcome in refractory acute myeloid leukemia patients treated with a combination of fludarabine, cytarabine, and amsacrine followed by a reduced-intensity conditioning and allogeneic stem cell transplantation. *J Cancer Res Clin Oncol* 2016; **142**. doi:10.1007/s00432-015-2050-y.

10	López-Ratón M, Rodríguez-Álvarez MX, Cadarso-Suárez C, Gude-Sampedro F. Optimalcutpoints: An R package for selecting optimal cutpoints in diagnostic tests. *J Stat Softw* 2014; **61**. doi:10.18637/jss.v061.i08.

11	Harris AC, Young R, Devine S, Hogan WJ, Ayuk F, Bunworasate U *et al.* International, Multicenter Standardization of Acute Graft-versus-Host Disease Clinical Data Collection: A Report from the Mount Sinai Acute GVHD International Consortium. *Biology of Blood and Marrow Transplantation* 2016; **22**: 4–10.

12	Fine JP, Gray RJ. A Proportional Hazards Model for the Subdistribution of a Competing Risk. *J Am Stat Assoc* 1999; **94**. doi:10.1080/01621459.1999.10474144.

13	R Core Team. R Core Team 2021 R: A language and environment for statistical computing. R foundation for statistical computing. https://www.R-project.org/. *R Foundation for Statistical Computing* 2022; **2**.

***Supplementary Tables***

**Table S1 - Main patients’ characteristics and patients’ characteristics according to relapse status**

|  |  | n (%) |  |  |
| --- | --- | --- | --- | --- |
| characteristics | all patients  (n = 106) | relapsed patients  (n = 69) | relapse-free patients (n = 30) | ***P*** |
| **Age at HSCT (years)** |  |  |  |  |
| median (range) | 55 (21-73) | 54 (21-73) | 57 (28-67) | .139 |
| **Patient sex** |  |  |  |  |
| female | 54 (51) | 34 (49) | 16 (53) | .828 |
| male | 52 (49) | 35 (51) | 15 (50) |  |
| **Disease** |  |  |  |  |
| *de novo* AML | 54 (51) | 38 (55) | 12 (40) | .194 |
| secondary AML | 52 (49) | 31 (45) | 18 (60) |  |
| **EMD** | 5 (5) | 2 (3) | 2 (7) |  |
| **Cytogenetics** |  |  |  |  |
| normal karyotype | 29 (27) | 18 (26) | 9 (30) | .508 |
| complex karyotype | 20 (19) | 12 (17) | 7 (23) |  |
| other aberrations | 43 (41) | 27 (39) | 13 (43) |  |
| na | 10 (9) | 8 (12) | 1 (3) |  |
| **Molecular genetics** |  |  |  |  |
| *NPM1* mut | 9 (16) | 4 (14) | 3 (15) | ≥.999 |
| *NPM1* wt | 46 (84) | 25 (86) | 17 (85) |  |
| **MRC risk** |  |  |  |  |
| favorable | 3 (3) | 1 (2) | 2 (3) | .468 |
| intermediate | 51 (53) | 33 (54) | 15 (52) |  |
| adverse | 42 (44) | 27 (44) | 12 (41) |  |
| **ELN2022** |  |  |  |  |
| favorable | 5 (8) | 2 (5) | 2 (11) | .656 |
| intermediate | 17 (26) | 12 (28) | 4 (21) |  |
| adverse | 45 (67) | 29 (67) | 13 (68) |  |
| **Blast count at diagnosis (%)** |  |  |  |  |
| median (range) |  |  |  |  |
| in peripheral blood | 22 (0-93) | 25,5 (1-90) | 22 (0-93) | .466 |
| in bone marrow | 40 (9-100) | 49 (9-95) | 36 (11-100) | .278 |
| **Prior treatment** |  |  |  |  |
| < 3 lines of prior treatment | 70 (66) | 39 (57) | 26 (87) | .005 |
| ≥ 3 lines of prior treatment | 36 (34) | 30 (43) | 4 (13) |  |
| prior allogeneic HSCT | 14 (13) | 10 (15) | 3 (10) | .749 |
| **Remission status prior to HSCT** |  |  |  |  |
| PIF | 64 (60) | 38 (55) | 23 (77) | .047 |
| 1st relapse | 36 (34) | 26 (38) | 6 (20) |  |
| 2nd relapse | 6 (7) | 5 (7) | 1 (3) |  |
| **Blast count prior to HSCT (%)** |  |  |  |  |
| in peripheral blood | 2 (0-94) | 2 (0-94) | 1 (0-34) | .018 |
| in bone marrow | 16 (4.6-95) | 17 (4.6-95) | 12 (6-50) | .044 |
| **time from diagnosis to HSCT** |  |  |  |  |
| median (range) | 6 (0-45) | 7 (1-43) | 3,5 (0-45) | .012 |
| **HCT-CI** |  |  |  |  |
| 0 points | 24 (27) | 15 (27) | 8 (31) | >.999 |
| 1-2 points | 23 (26) | 16 (29) | 7 (27) |  |
| ≥ 3 points | 41 (47) | 24 (44) | 11 (42) |  |
| **Conditioning** |  |  |  |  |
| 2Gy TBI | 2 (2) | 2 (3) | 0 (0) | .128 |
| BuCy | 16 (15) | 9 (13) | 6 (20) |  |
| CyTBI | 68 (64) | 49 (71) | 16 (53) |  |
| Mel | 13 (12) | 7 (10) | 5 (17) |  |
| TreoCy | 6 (6) | 1 (2) | 3 (10) |  |
| **Donor** |  |  |  |  |
| MSD | 9 (9) | 6 (9) | 3 (10) | .555 |
| MUD | 66 (62) | 43 (62) | 17 (57) |  |
| MMUD | 28 (26) | 19 (28) | 8 (27) |  |
| haplo | 3 (3) | 1 (1) | 2 (7) |  |
| **Stem cell source** |  |  |  |  |
| PBSC | 103 (97) | 67 (97) | 29 (97) | >.999 |
| BM | 3 (3) | 2 (3) | 1 (3) |  |
| **CD34+ cell/kg BW transplanted** |  |  |  |  |
| median (range) | 6.69 (1.01-29.1) | 7.1 (1.01-29.1) | 5.955 (3.4-14.8) | .079 |
| **Sex (recipient/donor)** |  |  |  |  |
| male/female | 14 (13) | 10 (15) | 4 (13) | >.999 |
| other | 88 (83) | 56 (81) | 26 (87) |  |
| **Blood type compatibility** |  |  |  |  |
| compatible | 51 (48) | 34 (49) | 14 (47) | .918 |
| minor incompatibility | 22 (21) | 13 (19) | 7 (23) |  |
| major incompatibility | 33 (31) | 22 (32) | 9 (30) |  |
| **CMV status (recipient/donor)** |  |  |  |  |
| CMV mismatch (IgG+/IgG-) | 40 (38) | 29 (42) | 8 (27) | .179 |
| **Toxoplasmosis status (recipient/donor)** |  |  |  |  |
| Toxoplasmosis mismatch (IgG+/IgG-) | 36 (34) | 24 (35) | 9 (30) | .817 |
| **time to engraftment (days)** |  |  |  |  |
| median (range) |  |  |  |  |
| leucocytes > 1 Gpt/l | 14 (8-49) | 14 (9-49) | 14 (8-26) | .729 |
| thrombocytes >50 Gpt/l | 15.5 (10-97) | 15 (10-97) | 19 (10-68) | .206 |
| **Remission status at day 28 ± 7 post HSCT** |  |  |  |  |
| refractory | 19 (18) | 16 (23) | 3 (10) | .703 |
| PR | 11 (10) | 8 (12) | 3 (10) |  |
| MLFS | 8 (8) | 5 (7) | 3 (10) |  |
| CRi | 28 (26) | 19 (28) | 9 (30) |  |
| CR | 20 (19) | 13 (19) | 7 (23) |  |
| na | 20 (19) | 8 (12) | 5 (17) |  |
| **Chimerism at day 28 ± 7 post HSCT** |  |  |  |  |
| **total** median (range) | 100 (0-100) | 100 (0-100) | 100 (14-100) | .614 |
| **CD34+** median (range) | 100 (0-100) | 100 (0-100) | NA | NA |
| **Remission status at day 100 ± 28 post HSCT** |  |  |  |  |
| relapse | 22 (21) | 21 (30) | 0 (0) | .003 |
| PR | 3 (3) | 2 (3) | 2 (7) |  |
| MLFS | 4 (4) | 2 (3) | 2 (7) |  |
| CRi | 11 (10) | 6 (9) | 5 (17) |  |
| CR | 20 (19) | 13 (19) | 7 (23) |  |
| na | 46 (43) | 25 (36) | 14 (47) |  |
| **Chimerism at day 100 ± 28**  **post HSCT** |  |  |  |  |
| **total** median (range) | 99 (0-100) | 99 (0-100) | 99 (61-100) | .310 |
| **CD34+** median (range) | 100 (0-100) | 100 (0-100) | NA | NA |
| **time of immunosuppressive therapy (days)** |  |  |  |  |
| median (range) | 109 (1-1064) | 109 (20-331) | 114 (26-1046) | .159 |
| **DLI** |  |  |  |  |
| received at least 1 prophylactic DLI | 13 (12) | 8 (12) | 5 (17) | .526 |
| **acute GvHD (MAGIC)** |  |  |  |  |
| °0 | 52 (49) | 35 (51) | 10 (33) | .055 |
| °I | 26 (25) | 19 (28) | 7 (23) |  |
| °II | 10 (10) | 7 (10) | 3 (10) |  |
| °III | 10 (10) | 6 (9) | 4 (13) |  |
| °IV | 8 (8) | 2 (3) | 6 (20) |  |
| **chronic GvHD** |  |  |  |  |
| documented | 17 (16) | 11 (15) | 6 (20) | .772 |

**Table S1 - Main patient's characteristics and patient's characteristics according to relapse status**

Note: For 7 patients, remission assessment was not possible after allo-HSCT due to early death in aplasia.

Abbreviations: **AML**, acute myeloid leukemia; **BM**, bone marrow; **Bu**, Busulfan, **BW**, bodyweight; **CMV**, cytomegalovirus; **CR**, complete remission; **CRi**, complete remission with incomplete hematologic recovery; **Cy**, Cyclophophamide; **DLI**, Donor Lymphocyte Infusion; **EMD**, extramedullary disease; **GvHD**, Graft-versus-Host-Disease; **haplo,** haploidentical; **HCT-CI**, hematopoietic cell transplantation-specific comorbidity index; **HSCT**, allogeneic hematopoietic stem cell transplantation; **MAGIC**, Mount Sinai Acute GvHD Consortium; **Mel**, Melphalan; **MLFS**, morphologic leukemia free state; **MMUD**, mismatch unrelated donor; **MSD**, matched sibling donor; **MRC**, medical research council; **MUD**, matched unrelated donor; **mut**, mutated; ***NPM1***, nucleophosmin1-gene; **PIF**, primary induction failure; **PR**, partial remission; **TBI**, total body irradiation; **Treo**, Treosulfan; **wt**, wild type.

**Table S2 – Factors without prognostic relevance in univariate analysis**

|  |  | **Univariate Analysis** | | |
| --- | --- | --- | --- | --- |
|  |  | **OS** |  | **EFS** |
| **characteristics** |  | ***P*** |  | ***P*** |
| Sex | *female vs. male* | .68 |  | .74 |
| Disease | *de novo vs. secondary AML* | .73 |  | .68 |
| ELN22 risk | *favorable vs. intermediate vs. adverse* | .86 |  | .86 |
| Months from Dx to HSCT | *≤ 6 months vs. >6 months* | .88 |  | .81 |
| Prior treatment lines | *< 3 vs. ≥ 3 lines of prior treatment* | .51 |  | .23 |
| Prior allo-HSCT | *none vs. ≥ 1 HSCT* | .84 |  | .76 |
| HCT-CI | *low (0 pts) vs. intermediate (1-2 pts) vs. high (≥3 pts)* | .49 |  | .25 |
| Donor | *MUD vs. MMUD vs. MSD vs. haploidentical* | .24 |  | .47 |
| Sex mismatch | *female donor into male patient vs. other* | .65 |  | .25 |
| Blood type compatibility | *match vs. minor mismatch vs. major mismatch* | .98 |  | .99 |
| Acute GvHD | *°0-I vs. °II-IV* | >.99 |  | .53 |
| Chronic GvHD | *none vs. any (landmark analysis from day 100)* | .18 |  | .24 |

**Table S2 - Factors without prognostic relevance in univariate analysis**

Characteristics with *P* >.2 for OS and EFS in univariate analysis were not included in further multivariate analyses.

*Abbreviations:* **AML**, acute myeloid leukemia; **Dx**, diagnosis; **ELN**, European LeukemiaNet; **GvHD**, Graft-versus-Host-Disease; **MMUD**, mismatch unrelated donor; **MSD**, matched sibling donor; **MUD**, matched unrelated donor

**Table S3 - Univariate analysis and multivariate analyses**

|  |  | **Univariate Analysis** | | |  | **Multivariate Analyses** | | | | |
| --- | --- | --- | --- | --- | --- | --- | --- | --- | --- | --- |
|  |  | **OS** |  | **EFS** |  | **OS** | |  | **EFS** | |
| **characteristics** |  | ***P*** |  | ***P*** |  | **HR (CI)** | ***P*** |  | **HR (CI)** | ***P*** |
| Age at HSCT | *21-41y vs. 42-54y vs. 55-61y vs. 62-73y* | .13 |  | .15 |  | 0.91 (0.76 - 1.1) | .265 |  | 0.89 (0.75 - 1.1) | .18 |
| Cytogenetics | *cKT & mKT &TP53 vs. other* | .11 |  | .053 |  | 1.43 (0.75 - 2.7) | .278 |  | 1.53 (0.74 - 3.2) | .256 |
| MRC risk | *favorable vs. intermediate vs. adverse* | .31 |  | .11 |  | x | x |  | 1.06 (0.58 - 1.9) | .851 |
| BM blast count at HSCT | *≤ 16% vs > 16%* | **.024** |  | **.026** |  | 1.91 (1.1 - 3.3) | **.021** |  | 2.22 (1.26 - 3.9) | **.006** |
| pB blast count at HSCT | *0% vs ≥ 1%* | .10 |  | .07 |  | 1.05 (0.56 - 1.9) | .882 |  | 0.95 (0.51 - 1.8) | .876 |
| Conditioning | *TBI Cy vs. Bu Cy vs. Mel vs. Treo Cy vs. 2GyTBI* | .27 |  | .12 |  | x | x |  | 1.21 (0.94 - 1.6) | .146 |
| CMV status | *all vs. high risk (IgG neg into IgG pos)* | .13 |  | .14 |  | 0.65 (0.38 - 1.1) | .136 |  | 0.61 (0.35 - 1.1) | .095 |
| Prophylactic DLI | *no DLI vs. ≥1 DLI* | .17 |  | .42 |  | x | x |  | x | x |

**Table S3 - Univariate analysis and multivariate analyses**

Characteristics with *P*<.2 for OS and/or EFS in univariate analysis were further included in the COX models for multivariate analyes for OS and EFS. BM blast count at allo-HSCT (<16% vs. ≥16%) retained its significance.

*Abbreviations:* **BM**, bone marrow; **Bu**, Busulfan; **CI**, Confidence Interval (0.95); **cKT**, complex karyotype; **CMV**, cytomegalo virus; **Cy**, Cyclophophamide; **GvHD**, Graft-versus-Host-Disease; **haplo,** haploidentical; **HCT-CI**, hematopoietic cell transplantation-specific comorbidity index; HR, Hazard Ratio; **HSCT**, allogeneic hematopoietic stem cell transplantation; **IgG**, Immunglobuline G; **Mel**, Melphalan; **mKT**, monosomal karyotype; **MRC**, medical research council; **pB**, peripheral blood; **TBI**, total body irradiation; **Treo**, Treosulfan.

***Supplementary Figures***

**Figure S1 - Outcome according to blast count in bone marrow prior to allo-HSCT**

**Figure S1 - Outcome according to blast count in bone marrow prior to allo-HSCT**

**A** Event-free survival of patients with ≤ 16% blasts *vs.* patients with > 16% blasts in bone marrow; **B** Overall survival of patients with ≤ 16% blasts *vs.* patients with > 16% blasts in bone marrow.

*Abbreviations:* **y**, years

**Figure S2 - Competing risk analysis for factors with significant outcome associations in univariate analysis**

**Figure S2 - Competing risk analysis for factors with significant outcome associations in univariate analysis**

Competing risk analyses on factors with significant outcome associations or trend to significance.

**A** Comparing patients with ≤ 6 months from diagnosis to HSCT *vs.* > 6 months prior to HSCT. **B** Comparing patients who received < 3 lines of therapy prior to HSCT to patients receiving ≥ 3 lines of therapy prior to HSCT. **C** Comparing patients with primary induction failure to patients, who underwent HSCT after first relapse to patients, who underwent HSCT after 2^nd^ relapse. **D** Comparing patients, who obtained blast clearance in peripheral blood to patients, who had persistent blasts in peripheral blood. **E** Comparing patients, who have less than 16% to 16% and more blasts in bone marrow prior to HSCT.

*Abbreviations:* CIR, cumulative incidence of relapse; Dx, diagnosis, HCT-CI, Hematopoietic Cell Transplantation-specific Comorbidity Index; NRM, non-relapse mortality; PIF, primary induction failure

**Figure S3 - Outcome according to remission status at day 28 post HSCT**

**Figure S3 - Outcome according to remission status at day 28 post HSCT**

**A** OS and **B** EFS of patients achieving cCR or MLFS compared to patients with persisting AML blasts in bone marrow at day 28 ± 7 after HSCT.

Abbreviations: **cCR**, composite complete remission; **MLFS**, morphologic leukemia-free state; PR, partial remission.

**Figure S4 – Survival according to conditioning prior to allo-HSCT**

**Figure S4 – Survival according to conditioning prior to allo-HSCT**

Kaplan-Meier estimates for **A** Overall survival and **B** Event-free survival.

*Abbreviations:* **Bu**, busulfane; **Cy**, cyclophosphamide; **Gy**, Gray; **Mel**, melphalane; **Treo**, treosulfane.

**Figure S5 - C statistics of bone marrow chimerism at different timepoints**

**Figure S5 - C statistics of bone marrow chimerism at different timepoints**

Area under the curves (AUC), calculated with **A** total bone marrow chimerism and **B** chimerism of sorted CD34+ cells for prediction of relapse within the next 84, 56, 28 and 14 days.

**Supplementary Figure S6 - Survival according to age at HSCT**

### **Supplementary Figure S6 - Survival according to age at HSCT**

Kaplan-Meier estimates for **A** OS and **B** EFS of all patients according to age. Patients were divided into age-quartiles.

**Figure S7 - Landmark Analysis at day 165 according to DLI**

**Figure S7 - Landmark Analysis at day 165 according to DLI**

Landmark analysis at day 165 (median time from allo-HSCT to DLI) for **A** OS and **B** EFS of patients receiving at least one prophylactic DLI compared to patients who did not receive prophylactic DLI.

*Abbreviations:* **DLI**, donor lymphocyte infusion
